# Assessing Learning‐Based Reconstructed Liver Surfaces From Partial Point Clouds for Improving Pre‐ to Intra‐Operative 3D to 3D Registration

**DOI:** 10.1049/htl2.70041

**Published:** 2025-12-09

**Authors:** Nakul Poudel, Zixin Yang, Richard Simon, Cristian A. Linte

**Affiliations:** ^1^ Center for Imaging Science Rochester Institute of Technology Rochester New York USA; ^2^ Biomedical Engineering Rochester Institute of Technology Rochester New York USA

**Keywords:** image‐guided liver surgery, registration, surface completion, uncertainty assessment

## Abstract

The registration process for fusing pre‐operative information with intra‐operative data collected during image‐guided liver surgery struggles due to partial visibility. Learning‐based partial point cloud‐to‐complete surface generation has shown a promising direction for improving registration outcomes. Yet, the intra‐operative liver surface can undergo significant deformation, leading to geometric discrepancies from its pre‐operative shape and introducing error in the completed intra‐operative surface. It is essential to understand the error introduced during surface generation and its impact on both rigid and non‐rigid registration to ensure robust performance in clinical settings. In this study, we leveraged a VN‐OccNet framework trained in a patient‐specific manner on simulated deformed data to generate complete surfaces from partial observations extracted from five viewpoints across four in vitro liver phantoms. We first analysed the error associated with the generated complete surface mesh from the partial point cloud, then integrated the complete surface generated into Go‐ICP and GMM‐FEM registration. Furthermore, we estimated the registration error separately for visible and invisible regions. Our results indicate that the error in the generated surface is more significant further away from the partially visible liver surface, and it could affect the registration, not only in the invisible region, but to some extent also in the visible region within the camera's field of view.

## Graphical abstract

This study investigates the impact of surface generation error on registration accuracy during image‐guided liver surgery. Using a patient‐specific VN‐OccNet trained on simulated deformations, complete liver surfaces were reconstructed from partial intra‐operative point clouds and then registered using Go‐ICP and GMM‐FEM. Results show that surface generation errors increase with distance from the visible area and can impact both visible and invisible regions during registration.

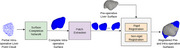

## 1 Introduction

Pre‐operative imaging modalities commonly employed for minimally invasive liver surgery involve magnetic resonance imaging (MRI) and computed tomography (CT), which provide high‐resolution volumetric information. However, because of the high acquisition costs, prolonged imaging time and overall impractical intra‐procedural use due to their large footprint in the interventional suite, the intra‐operative usage of these modalities is limited. As a result, the imaging data captured during image‐guided interventions relies on modalities such as stereo video cameras and optical tracking. These modalities capture intra‐operative surfaces without volumetric information. This limitation is crucial in liver surgery, as key regions of interest, such as tumours and vessels, lie beneath the surface.

The data captured during the pre‐operative and intra‐operative settings from different modalities can be represented in a common data format, such as a point cloud or mesh [1, 2] and 3D–3D registration can be performed for determining spatial correspondence between the pre‐ and intra‐operative data, allowing surgeons to localize the surgical target accurately. However, these registration methods [2, 3] struggle due to the partial visibility of the intra‐operative surface, which arises from the constrained camera viewpoint and occlusion [3, 4].

To address the issue of partial visibility, learning‐based reconstruction of the complete liver surface from a partial point cloud has been explored [4, 5, 6]. Jia et al. [4] utilized an occupancy network (OccNet [7]) to reconstruct the complete intra‐operative liver surface from a sparse point cloud, thereby guiding non‐rigid registration. Their proposed solution relies on an initial rigid registration to bring the intra‐operative points into a known pose, allowing the occupancy network to function. Poudel et al. [6] utilized a vector‐based method for occupancy networks (VN‐OccNet [8]), an extension of the occupancy network that is robust to rotations of the intra‐operative point cloud and avoids the need for initial alignment. However, to the best of our knowledge, no prior work has investigated the error associated with learning‐based reconstructed surfaces and its effect on rigid and non‐rigid registration algorithms.

In this work, we utilize four in vitro phantoms to study the error associated with the complete surface generation from VN‐OccNet, a learning‐based model trained in a patient‐specific manner on liver surfaces simulated under various deformations. As the liver is a complex anatomical structure and possesses considerable inter‐patient variability [9, 10], a patient‐specific approach enables a learning‐based method to focus on complex geometric and deformation patterns specific to each liver. Additionally, the pre‐operative liver image and hence the inherent liver model is acquired a few days prior to surgery, making it practical to simulate deformations and develop a patient‐specific completion model.

To determine how the error impacts the registration, we integrated the patches extracted from the generated complete surface into both rigid (Go‐ICP [11]) and non‐rigid (GMM‐FEM [12]) registration algorithms. The regions of the liver that are within the camera's field of view are of particular clinical relevance to the surgeon [13]. To better understand how surface completion impacts registration in these critical areas, we compute the registration error separately for the visible and invisible regions of the liver, as defined by the camera's field of view.

## 2 Methodology

### 2.1 Overview

**FIGURE 1:**
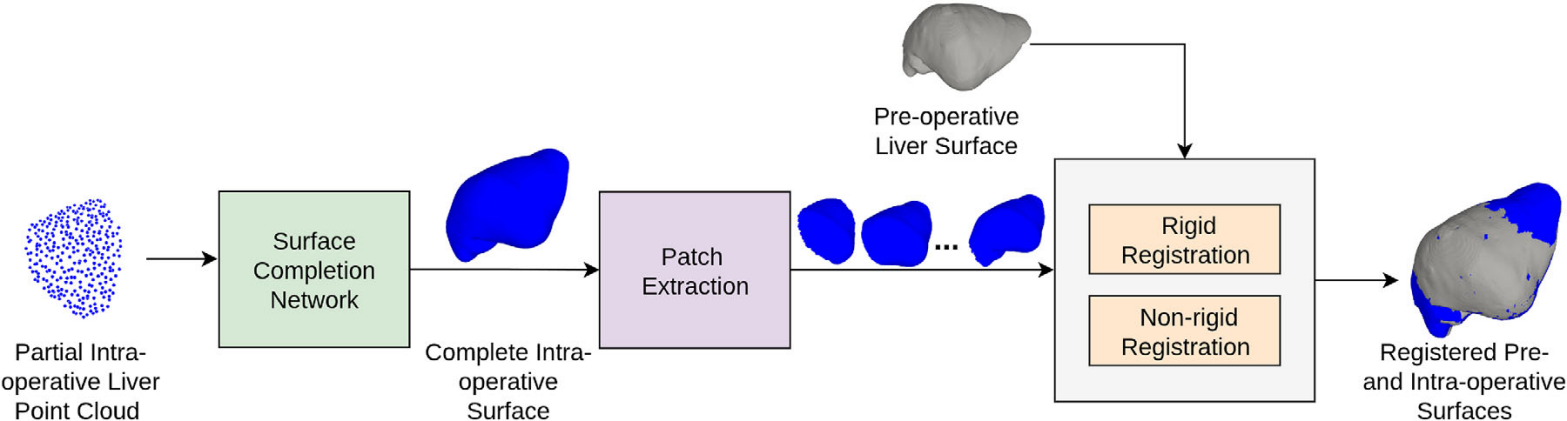
Our experiment pipeline. The complete liver surface is generated from a partial point cloud using a surface completion network. The registration is performed between the patches extracted from the complete generated surface and the pre‐operative liver surface.

The overall pipeline of our experimental procedure is shown in Figure 1. The partial point cloud obtained during image‐guided surgery is passed to the surface completion network to generate the complete intra‐operative surface, from which surface patches at various coverages are extracted and then passed to the registration algorithm. Due to the integration of water‐tight mesh generation and the only surface completion algorithm that possesses a rotational‐equivariance property, we leverage VN‐OccNet, a modification of OccNet [7], for complete surface generation. VN‐OccNet is an encoder‐decoder based network that extends the scalar representation of neurons to 3D vectors. The encoder is based on the PointNet network [14], which takes the partial liver point cloud as input and outputs the latent vector‐list features. The query point and latent features are input to the decoder network, which outputs the occupancy probability between 0 and 1. The probability value greater than or equal to some threshold c implicitly represents the presence of a surface point. The integrated mesh generation pipeline includes the multiresolution isosurface extraction (MISE) [7] algorithm, which identifies surface‐intersecting voxels using the trained VN‐OccNet. All surface‐intersecting voxels are then passed to the marching cube algorithm [15] to construct the complete mesh.

To test how the error in the reconstructed surface influences the outcome of rigid and non‐rigid registration, we extracted patches at different coverages and performed registration between surface patches extracted from the generated complete surface and the pre‐operative surface. We utilize Go‐ICP for rigid registration and GMM‐FEM for non‐rigid registration. As Go‐ICP requires the surface in the form of point clouds, we extracted the vertices of the patches and the pre‐operative surface to perform registration. In contrast, GMM‐FEM directly leverages the mesh structure for non‐rigid alignment. For clarity, the pre‐operative liver surface is referred to as the **source** and the intra‐operative liver surface as the **target**. The target appears in four forms: the partial target (partially visible intra‐operative point cloud), the generated target (generated complete intra‐operative surface), the target patch (patch extracted from the generated surface) and the ground truth target (complete ground truth deformed surface). Since the core of this framework is a surface generation network, it is expected that an error‐free generated surface leads to a better registration outcome.

### 2.2 Dataset

**FIGURE 2:**
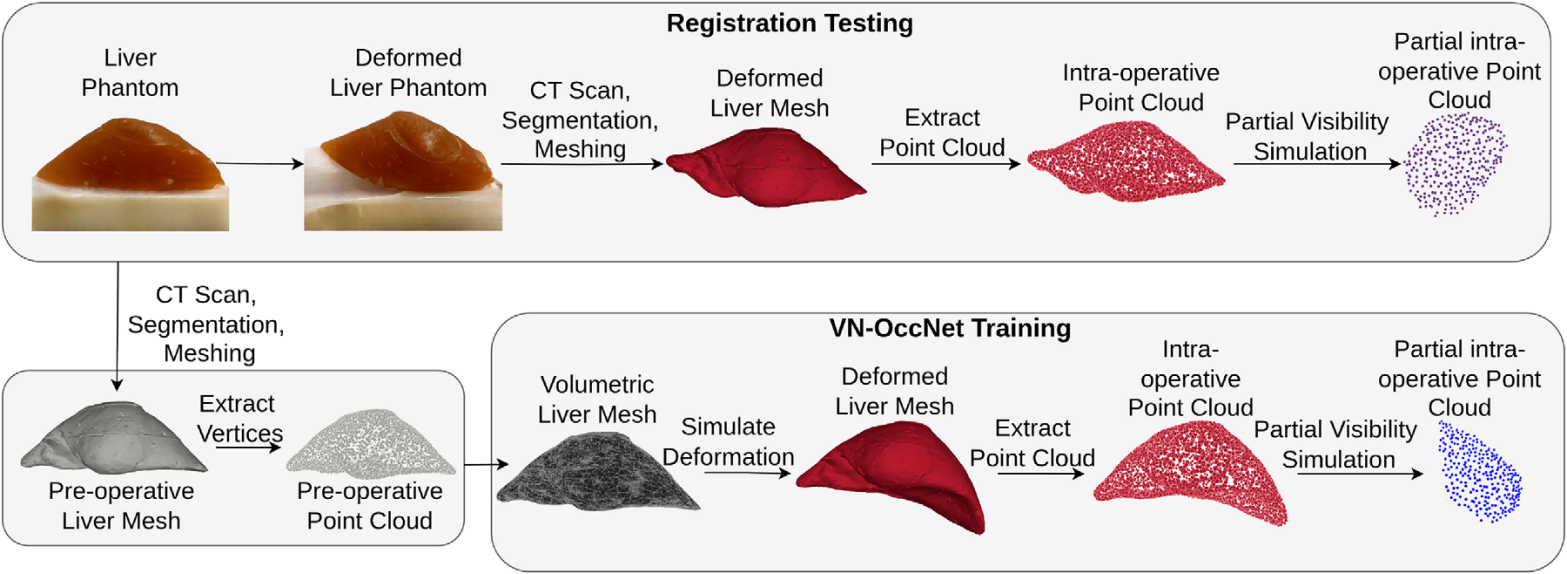
Data generation pipeline. in vitro phantoms are used to assess registration performance, while simulated deformations generate in silico phantoms for training and testing VN‐OccNet.

The experiments utilized two different datasets: the in silico phantom dataset to train and test VN‐OccNet and the in vitro phantom dataset to assess the registration performance, as shown in the data generation pipeline in Figure 2.

#### 2.2.1 in vitro Phantoms

**FIGURE 3:**
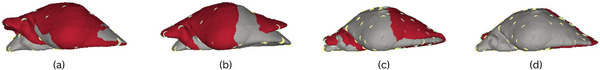
The gray phantoms in (a)–(d) represent undeformed phantoms No. 1–4, respectively, while the red phantoms represent their ground truth deformed versions.

Four in vitro phantoms developed and released in our previous work [16] are utilized. First, an undeformed liver phantom is created by printing a 3D mould [17] based on a patient‐specific liver model from OpenHelp [18] and pouring synthetic gelatin (Humimic Gelatin # 0, Humimic Medical, Fort Smith, AK, USA) into the generated mould. Four deformed phantoms numbered (No. 1–4) as shown in Figure 3, are obtained by placing wedges underneath at different regions of the undeformed liver phantom. For phantoms No. 1, 3 and 4, the wedge is placed under the left lobe; for phantom No. 2, the wedge is positioned under both lobes of the liver. The regions directly deformed by the wedge exhibit noticeable deformation, whereas the other areas show a lesser extent of deformation. Among all, phantoms No. 1 and 2 featured larger deformation, and phantoms No. 3 and 4 featured more moderate deformation. Phantom No. 2 exhibits a complex structure due to bilateral lobe involvement. Phantoms No. 1 and 2 feature 53 fiducial markers, while Phantoms No. 3 and 4 feature 176 fiducial markers.

The undeformed liver phantom constitutes the pre‐operative data, and the deformed liver phantom constitutes intra‐operative data. CT scans of both deformed and undeformed liver phantoms are captured, and surface and fiducial marker locations are segmented. The partial visibility pipeline, outlined in the following subsection, is followed to simulate partial intra‐operative visibility. Since both pre‐operative and intra‐operative data are generated from CT scans and subsequent segmentation, small errors may be introduced during this process.

#### 2.2.2 in silico Phantoms

The in silico phantoms are generated by simulating the deformations of an undeformed liver model following [19]. A total of 4969 deformed liver models are generated, which are divided in the ratio 8:1:1 for training, validation and testing of VN‐OccNet. As the training of the occupancy network requires data consisting of point clouds, occupancy points and query points, we followed the pipeline of Stutz et al. [20] to generate this data format.

### 2.3 Partial Visibility Simulation

**FIGURE 4:**
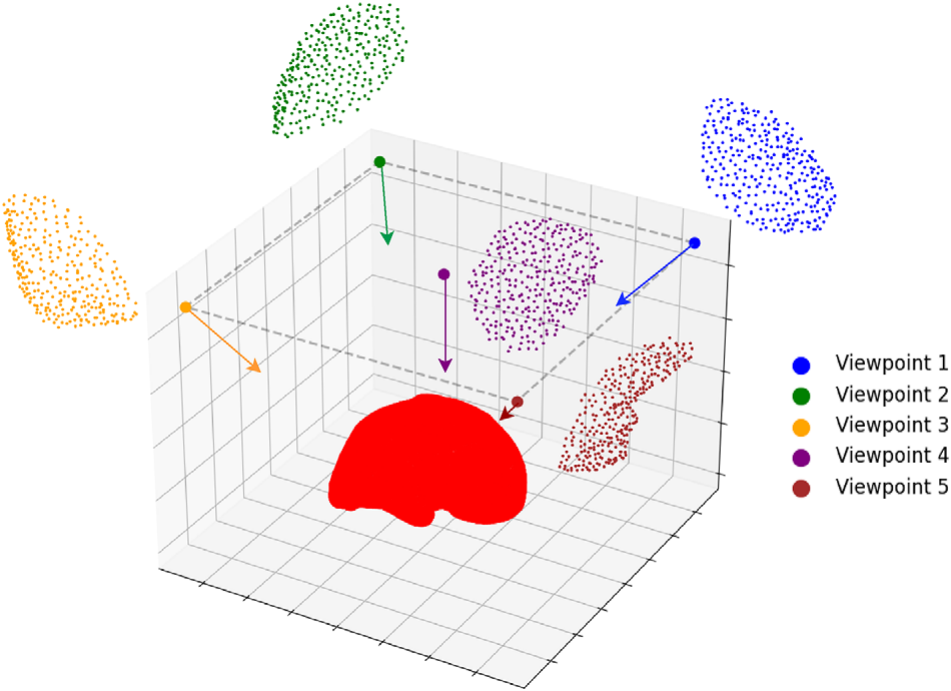
Five partial target point clouds generated from five unique viewpoints to mimic partial visibility. In this example, each partial point cloud represents approximately 30% coverage of the anterior liver surface.

The partial target point clouds are generated from complete ground truth target point clouds following [6, 21]. First, the posterior surface of the liver is removed using a slicing plane. The anterior surface is downsampled to 1000 points, then 300 nearest points relative to the viewpoint are selected. During the training of VN‐OccNet, viewpoints are randomly generated. Five distinct viewpoints are used in the VN‐OccNet testing and registration evaluation, as shown in Figure 4. The surface visibility of partial targets is approximately 30% of the anterior liver surface. Viewpoint 1 captures the front part of the right lobe, while viewpoint 2 depicts its back side. Viewpoint 3 covers the left lobe, with most of the region visible from the back. Viewpoint 4 represents the central region of the liver, and, lastly, viewpoint 5 corresponds to the front part of the left lobe without overlapping with the front part of the right lobe.

### 2.4 Registration Setup

**FIGURE 5:**
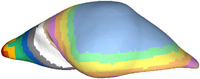
Visualization of patch extraction from the generated target. The initial light blue patch corresponds to the initial partial target. At each step, new patches indicated by different colours are added to expand the surface coverage progressively.

Registration is performed between the source and the target patches, representing various coverage levels extracted from the generated target from VN‐OccNet. The initial patch corresponds to the region captured by the initially acquired partial target, and the subsequent patches progressively expand to include areas further from the initial patch, gradually covering more of the completed surface until the entire surface is covered, as shown in Figure 5. As the source and target are approximately aligned, we apply 10 random rotations between $\left[-\frac{\pi }{2},\frac{\pi }{2}\right]$ to the target patches to simulate different initial positions between the source and target patch for rigid registration. For non‐rigid registration, the target patches are used without any transformation.

### 2.5 VN‐OccNet Implementation

VN‐OccNet is implemented following the PyTorch implementation of the neural implicit reconstruction framework by Deng et al. [8]. The network is trained with a batch size of 8 and a learning rate of 0.0001, using the Adam optimizer for 604 epochs on an NVIDIA A100 GPU. Based on the validation set, the optimal threshold value c is determined to be 0.4.

### 2.6 Evaluation

To assess and visualize the surface reconstruction error, we compute the distance from each vertex of the generated target to the nearest vertex in the ground truth target. Let P and G be the vertex sets of the generated target and ground truth target meshes, respectively. For each p∈P, d(p,G) computes the distance to the nearest vertex in G,

(1) $$d\left(p,G\right)=\min_{g\in G}∥p-g∥.$$

Similarly, the target registration error (TRE) is utilized to measure the misalignment between the registered pre‐operative (source) fiducial markers and the corresponding intra‐operative (target) fiducial markers.

(2) $$\text{TRE}=\frac{1}{N}\sum_{i=1}^{N}∥x_{i}-x_{i}^{\prime }∥,$$

where N is the number of fiducial markers, $x_{i}$ is the 3D position of the target ith fiducial marker, $x_{i}^{\prime }$ is the registered position of the ith source fiducial marker, and ∥·∥ denotes the Euclidean norm (L2 norm).

## 3 Results

### 3.1 Complete Surface Reconstruction Error Assessment and Visualization

**FIGURE 6:**
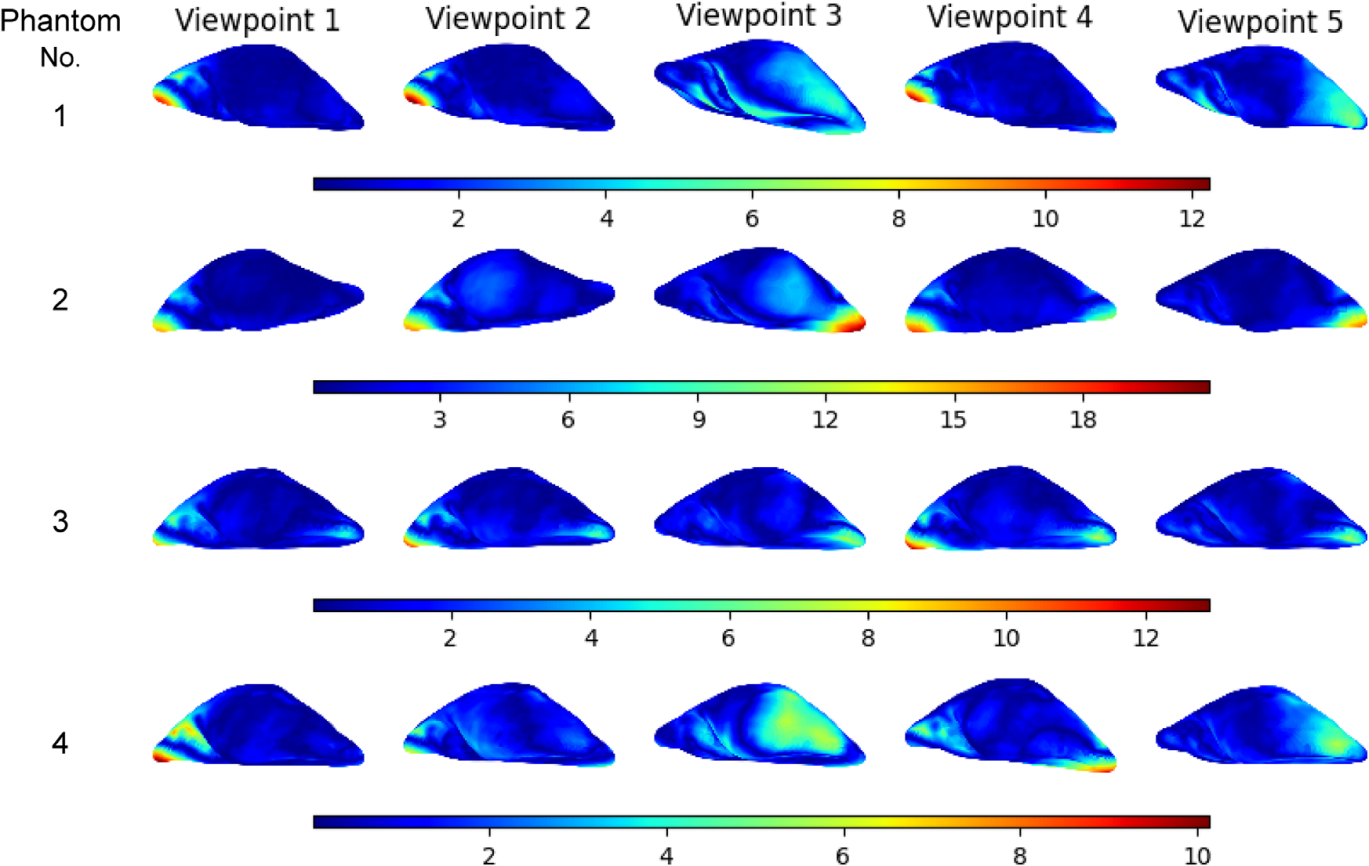
Visualization of generated targets for four phantoms with partial targets captured from five different viewpoints. The error bar shows the error in mm, measured as the Euclidean distance from each vertex in the generated target mesh to the nearest vertex in the ground truth target mesh.

The generated targets, obtained for four phantoms from five different partial targets captured from five unique viewpoints, are shown in Figure 6. The Euclidean distance from each vertex in the generated target to the nearest vertex in the ground truth target is measured to compute the error and visualized using a jet colour map. For each phantom, regions corresponding to the partial target consistently show minimal error (blue). In contrast, the error is high (red) in areas far from the partial target or in areas that are invisible. The situation worsens when a complex deformation pattern occurs in the invisible areas, as evident in the generated targets obtained from partial targets captured from viewpoints 1, 2 and 4 for phantoms No. 1, 3 and 4. When deformation is present in the visible region (partial targets captured from viewpoints 3 and 5), the generated target successfully captures the deformation in that region; however, it still exhibits error in the invisible areas. For phantoms No. 1, 3 and 4, the error falls within a similar range. In contrast, for phantom No. 2, the error is significantly higher compared to the other phantoms due to the complex deformation patterns, which posed challenges for the model to generate a correct surface.

### 3.2 Impact of Surface Generation Error on Target Registration Error

**FIGURE 7:**
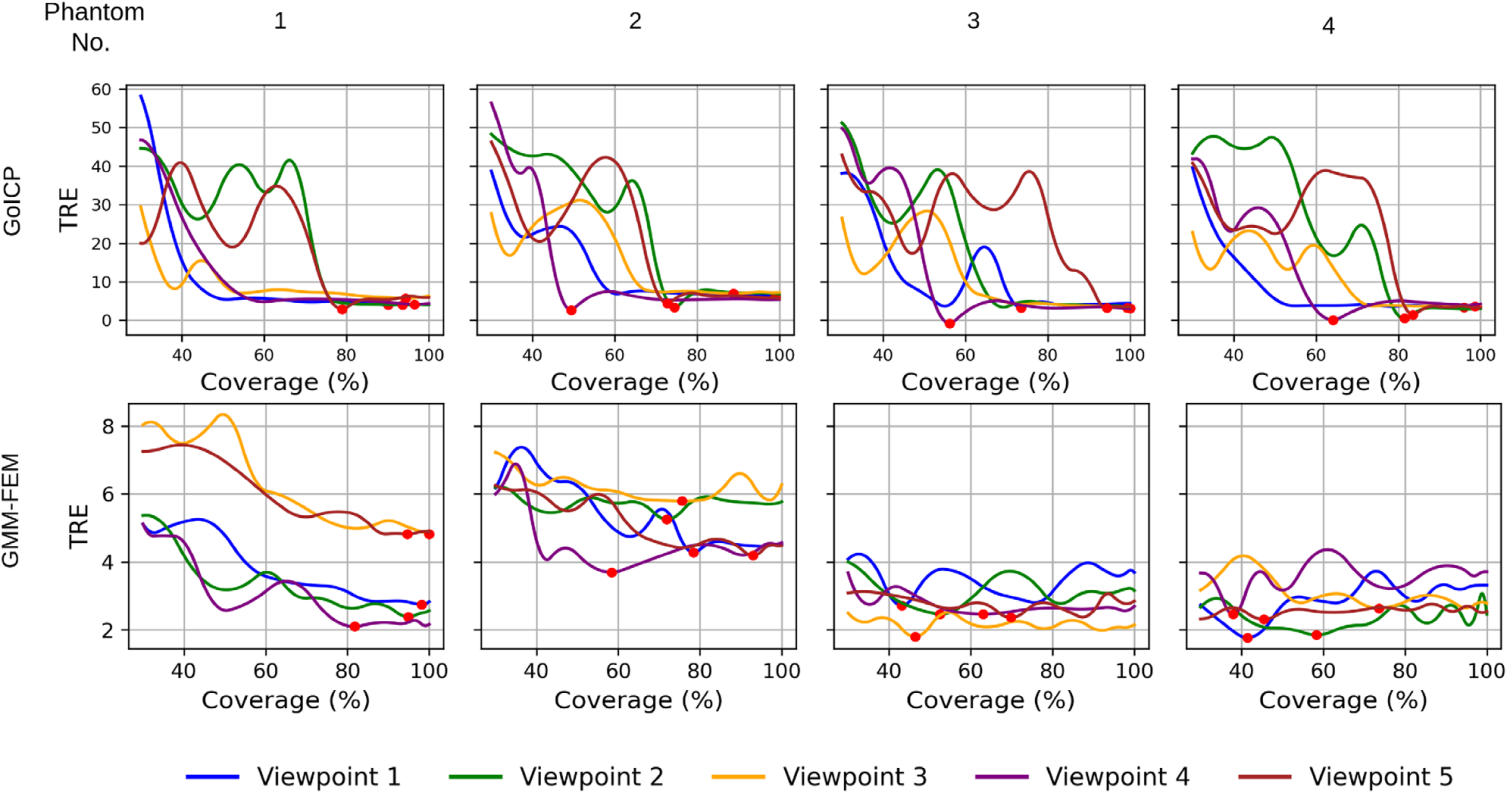
Target registration error (TRE in mm) versus surface coverage for four phantoms across five different partial targets extracted from five different viewpoints. The first row corresponds to the Go‐ICP rigid registration, while the second row corresponds to the GMM‐FEM non‐rigid registration. Red dots indicate the coverage where the optimal TRE is obtained.

Our surface reconstruction error estimation suggests that the completion error is higher in regions farther from the partial target. We now test how the error in surface generation propagates to both rigid and non‐rigid registration. We perform the registration between the target patches at various coverage and the source. The initially extracted target patches, being closer to the partial target, are more accurate, and as coverage increases, errors are introduced. Target registration errors (TRE) observed for various surface coverages across four phantoms, using five different partial targets, are shown in Figure 7 for both rigid (Go‐ICP) and non‐rigid (GMM‐FEM) registration.

For rigid registration, the TRE is very high at lower coverages, as the registration algorithm could not determine the correct correspondence based only on a small target patch. The TRE significantly reduces at higher surface coverage. We note that optimal TRE (indicated by a red dot) is not achieved at full coverage for most viewpoints, due to error present in the generated targets that misguides the registration process. For phantoms, No. 2, 3 and 4, viewpoint 4, which shows reconstruction error on both the left and right sides of the generated target, achieves the optimal TRE at much lower coverage than other viewpoints.

For non‐rigid registration, we observe a similar trend of reduction in TRE with increasing surface coverage, with optimal TRE achieved below full coverage. Phantom No. 1 shows a large TRE for viewpoints 3 and 5, both of which capture deformation present on the liver. The model is able to capture deformation, but the larger portion of the invisible region is erroneous, and this has propagated to the registration. While other viewpoints for phantom No. 1 capture the non‐deformed regions, the visible area is predicted accurately, with some error only present in a small section of the invisible region. Phantom No. 2, which exhibits the most complex deformation pattern among all phantoms, shows a large TRE due to the larger error present in the generated target surface. As phantom No. 2 features deformation on both lobes, all viewpoints except viewpoint 4 exhibit deformation, and viewpoint 5 somehow features more information on the deformation of both sides, even though it does not extend up to the right lobe to capture deformation on the right lobe. The generated targets from viewpoints 2 and 3 show larger error regions, and, consequently, lead to higher TRE when used in the registration. For all other viewpoints, the TRE is more or less similar at full coverage.

For phantoms No. 3 and No. 4, the deformation is relatively small compared to that of phantoms No. 1 and No. 2. For phantom No. 3, viewpoint 3, which captures deformation information, shows a small error in surface completion, which translates to a low TRE. However, viewpoints 1 and 2, which do not capture deformation, show large error in surface generation and a large TRE. For phantom No. 4, viewpoints without deformation coverage show a mixed range of TRE values. Viewpoints 3 and 5, which capture deformation, exhibit an error corresponding to a large invisible region, resulting in a higher TRE than that of viewpoint 2, which does not capture deformation.

These experiments suggest that the error in the generated target surface leads to an error in the registration result, regardless of whether the partial target captures deformation or not. The surface generation may be incorrect in the invisible region without deformation when a partially target consists of deformation, and vice versa.

While acknowledging the error present in the reconstructed surfaces, we are interested in further examining whether these erroneous surfaces are of any use and if they could still provide some guidance to assist with better registration. To answer this question, we will focus on the optimal coverage achieved for each viewpoint in each phantom and examine the optimal coverage at different coverage levels for different phantoms.

**FIGURE 8:**
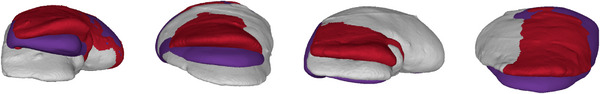
Visualization of the source mesh (grey), ground truth target mesh (red) and generated target mesh (purple) for phantoms No. 1, 2, 3 and 4, shown sequentially from left to right.

Figure 8 shows the source (grey), ground truth target (red), and generated target (purple) obtained using viewpoint 1 for phantoms No. 1‐4, respectively. For phantoms No. 1 and No. 2, we can see that the generated target is closer to the ground truth target; however, for phantoms No. 3 and No. 4, the source is closer to the ground truth. Even though an error is present in the generated target in phantoms No. 1 and 2, the generated target guides the registration towards the ground truth, and the best TRE is obtained at a large coverage. However, for phantoms No. 3 and 4, the generated target is far from the source, so it contributes negatively to that region when used for registration. Therefore, the best TREs for phantoms No. 3 and 4 are achieved at a much lower coverage that avoids those regions. In general, the best result can be achieved by considering all regions of the generated target that are closest to the ground truth target.

### 3.3 Registration Error on Visible and Invisible Regions

The visible region in the camera's field of view is the region of interest during surgery, and the registration outcome is critical for this region. We aim to determine how the registration error varies between visible and invisible regions and whether the optimal TRE obtained at the respective coverage (indicated by red dots in Figure 7) is due to improvements in visible or invisible areas, or both. We define a threshold of 30 mm from the centroid of the partial target point cloud and consider fiducial markers within this threshold to belong to the visible region. The remaining markers are considered to fall into the invisible region. Tables 1 and 2 summarize the registration outcome for four phantoms averaged across five viewpoints for both the visible and invisible regions for best and full coverage. The term full is used to denote the TRE at 100% coverage, and the term best is used to denote the optimal TRE, which, depending upon the phantom and viewpoint, can occur at a coverage less than 100% as depicted by the red dot in Figure 7 for each phantom and various viewpoints. Since the best coverage occurs below 100%, it avoids regions far away from the partial target where significant reconstruction errors are present.

**TABLE 1:** Average TRE (in mm) across five viewpoints for four phantoms using Go‐ICP registration. Results are reported for best and full surface coverage, with TRE values presented for fiducial markers in the visible region (≤30 mm from the partial target centroid) and fiducial markers in the invisible region (>30 mm from the partial target centroid).

	Visible		Invisible	
Phantom	Best	Full	Best	Full
1	3.77±2.22	3.95±2.25	4.98±3.24	5.10±3.25
2	4.01±1.08	4.09±1.13	6.63±5.05	6.63±4.95
3	2.59±1.33	2.60±1.34	3.66±2.06	3.60±2.08
4	2.71±0.69	2.74±0.69	3.68±1.67	3.71±1.65

**TABLE 2:** Average TRE (in mm) across five viewpoints for four phantoms using GMM‐FEM registration. Results are reported for best and full surface coverage, with TRE presented for fiducial markers in the visible region (≤30 mm from the partial target centroid) and fiducial markers in the invisible region (>30 mm from the partial target centroid).

	Visible		Invisible	
Phantom	Best	Full	Best	Full
1	2.37±1.51	2.40±1.52	3.58±2.60	3.65±2.65
2	2.51±1.15	3.08±1.03	5.04±4.23	5.45±4.28
3	1.66±0.91	2.02±1.23	2.54±1.69	3.11±2.08
4	1.80±0.53	2.07±0.81	2.31±1.33	3.13±1.92

As documented earlier in this manuscript, the error in surface generation is significant in regions farther away from the partial target, that is, in the invisible region. Moreover, we also observed large TRE in the invisible region for both rigid and non‐rigid registration, for both best and full coverage. Hence, we can conclude that erroneous surface generation in the invisible region leads to a poor registration result in that region.

For rigid registration, there are small improvements between the best and full coverage cases in both visible and invisible regions. Mainly, when the best case is achieved in the lower coverage range (phantoms No. 2, 3 and 4), the improvement is very slight. Therefore, for rigid registration, small coverage is not particularly helpful. However, for non‐rigid registration, we observe a notable improvement in TRE value, particularly for phantoms No. 2, 3 and 4, where the best registration accuracy is achieved at a lower coverage range.

The Best coverage, where the target patches are below 100% coverage and avoid regions far away from the partial target, where large reconstruction errors are likely, leads to lower registration errors, not only in the invisible areas, but in the visible areas. Therefore, for better TRE, achieving better surface completion in both visible and invisible regions is necessary.

#### 3.3.1 Threshold Sensitivity Study

Furthermore, we examined how varying the threshold value influences the TRE in the visible and invisible regions, as defined by each threshold, for both best and full coverage. As we increase the threshold, the visible region expands, and the invisible regions narrow.

**FIGURE 9:**
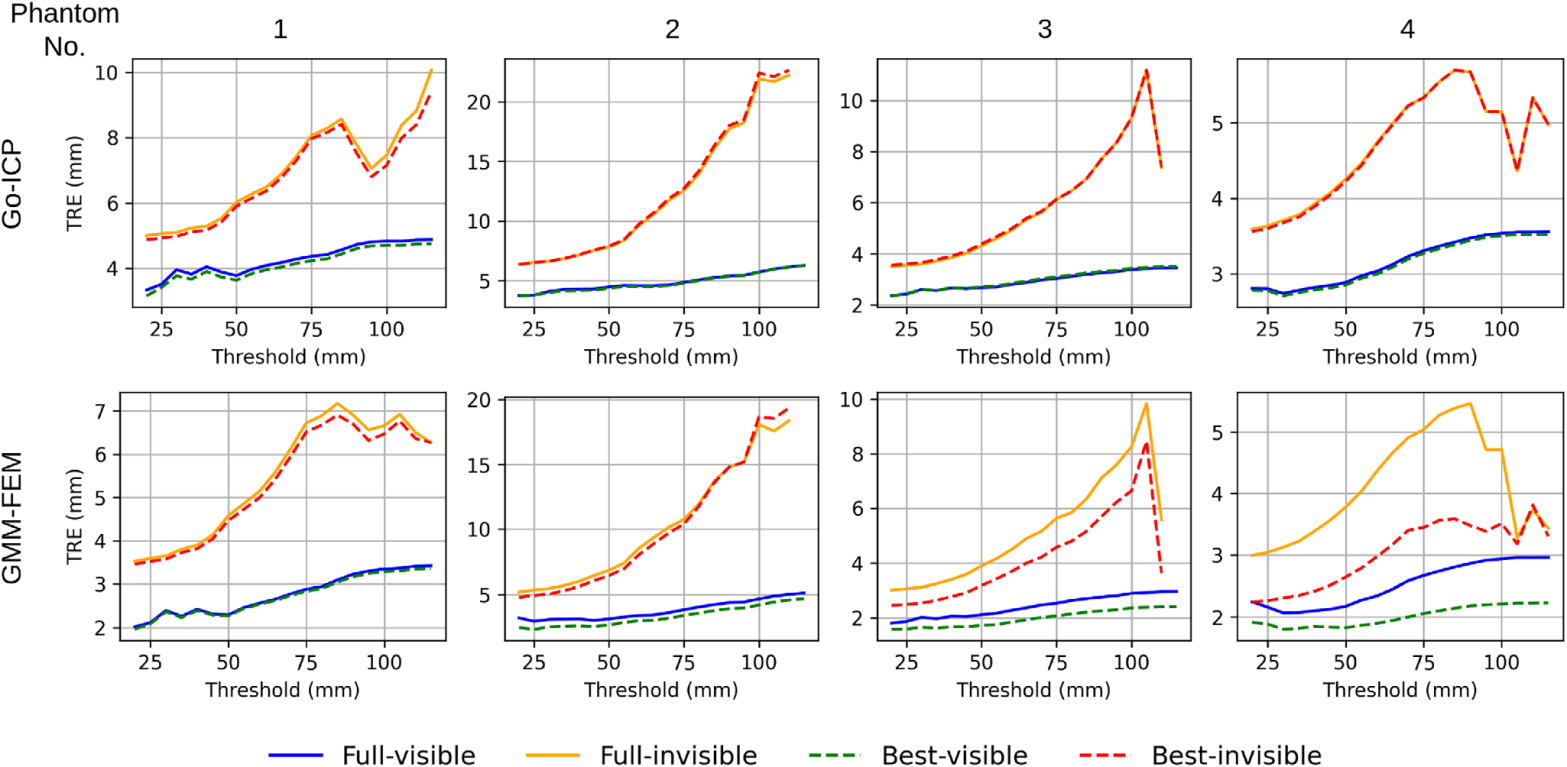
Sensitivity analysis plot. The threshold is varied, and visible and invisible regions are defined for each threshold value to measure TRE for both best and full coverage for Go‐ICP (first row) and GMM‐FEM (second row) registration.

From the previous subsection, we conclude that: (1) TRE is large in the invisible region for both rigid and non‐rigid registration. (2) For rigid registration, the differences between best and full coverage are not significant in either the visible or invisible regions, whereas for non‐rigid registration, noticeable improvements are observed between the visible and invisible regions for both best and full coverage. (3) For non‐rigid registration, best coverage provides lower TRE than full coverage in both the visible and invisible regions. These conclusions remain valid even when the threshold value is varied, as depicted by the sensitivity analysis summarized in the plot in Figure 9.

The dip seen in TRE in the invisible region at higher thresholds can be attributed to the presence of fewer fiducial markers.

## 4 Discussion

To address the issue of partial visibility of intra‐operative surfaces, which reduces the accuracy of both rigid and non‐rigid registration algorithms, learning‐based surface completion has demonstrated significant potential by generating a complete intra‐operative surface from partial observations. Past work leveraging learning‐based completion either requires manual interaction [13] or an initial alignment step [4]. Recent work [6] removes such dependencies by utilizing VN‐OccNet, a learning‐based technique that is robust to the rotation of partial intra‐operative surfaces. The integration of learning‐based methods holds strong promise; however, the liver surface, which can undergo significant deformation, and learning‐based methods, which assume the test data distribution follows that seen during training, may cause error in the generated surface. Understanding and analysing the error is crucial for developing a reliable and robust registration pipeline that leverages surface completion models. In this work, we utilize VN‐OccNet to generate surfaces from simulated partial intra‐operative point cloud data and analyse the error associated with the generated surface, as well as the impact of this error on both rigid and non‐rigid registration. Furthermore, we analysed the registration outcomes in the visible and invisible regions.

First, we analysed the error present in the complete surfaces generated using VN‐OccNet, which was trained in a patient‐specific way on simulated patient‐specific deformed liver data. We utilized four in vitro phantoms with realistic deformation imposed by placing a wedge underneath the posterior side of the phantom. Our partial view simulation pipeline generated five partial surfaces per phantom, capturing various regions of the liver. The error analysis conducted on the complete surfaces revealed that the higher surface reconstruction uncertainty corresponded to the surface regions farther from the partially visible surface. The regions of the reconstructed surface near the visible partial surface, regardless of the extent of deformation present, yielded higher accuracy than the regions located farther.

Secondly, we performed an analysis to better understand how the error present in the generated surfaces impacts the outcome of rigid and non‐rigid registration. We extracted patches from the generated surface. The first patch corresponded to visible region depicted by the partial surface initially used to reconstruct the complete surface. We then expanded the patch size to incorporate invisible areas made available by the complete surface reconstruction. Registration was then performed between the patches extracted from the generated surface and the source mesh. Our analysis suggested that initial patches led to poor performance for both the rigid and non‐rigid registration, with the impact of lower visibility being greater (i.e., less accurate alignment) for the rigid registration. Since non‐rigid registration relies on accurate initial alignment [22] for precise deformation estimation, poor rigid alignment can further degrade the performance of non‐rigid methods. Optimal registration was observed for partial coverage for most generated surfaces, indicating that the error present in the invisible regions of the reconstructed surfaces eroded the registration performance. Nevertheless, even if the generated complete surfaces were prone to error, they could still provide meaningful guidance for registration provided their overall shape was sufficiently representative of the actual intra‐operative surface deformation. This was observed in phantoms No. 1 and 2, where accurate registration was achieved at higher coverage levels, despite some errors in the generated complete surface. However, phantoms No. 3 and 4, for which the predicted deformed/intra‐operative surface differs significantly from the actual/ground truth intra‐operative surface, the usefulness of the generated complete surface became limited. While making this recommendation, we nevertheless acknowledge the inherent limitation that in a real clinical setting, a true intra‐operative deformed surface mesh is not available, and hence a predicted deformed intra‐operative mesh is generated and used instead.

Thirdly, considering that the visible intra‐operative surface is typically the region of interest for surgeons, we analysed how the error in the generated surface, which is more pronounced in the invisible region, impacts registration in both the visible and invisible areas. Using a threshold of 30 mm, we classified all fiducial markers within the 30 mm range as visible and the remaining markers as invisible. Our study suggested that the erroneous region in the complete surface generation, which is primarily located in the invisible region, affects the registration outcome in both the visible and invisible regions. Therefore, a better registration result requires an overall good surface, not just good surface generation in the visible region. Additionally, this analysis suggests that an error in the complete surface generation may not significantly impact rigid registration, but it substantially affects non‐rigid registration.

Our study may provide meaningful guidance for future research on developing a registration framework that leverages the surface completion network. However, there are several limitations to our current work. Our current study relies on a pipeline for generating clean, partially visible surfaces. The real intra‐operative data may involve a more complex structure of partial data, involving holes, noise and occlusion due to bleeding and the presence of surgical instruments. Understanding the impact of such factors on surface completion performance is the focus of our future work. In addition, this study utilizes in silico data to train the surface completion network and in vitro data consisting of patient‐specific emulating phantoms featuring the same patient as the in silico data to test the surface generation and registration. Evaluating the generalizability of the surface completion method to data from different patients remains an important direction for future investigation. Nonetheless, the integration of surface generation into the pipeline shows strong promise for improving tasks that are hindered by partial visibility, such as registration. At the same time, our analysis emphasizes the need for caution when using the generated surfaces, particularly in non‐rigid registration, which is sensitive to surface reconstruction accuracy.

## 5 Conclusions

In conclusion, we investigated the error associated with the generated surfaces from the VN‐OccNet network, aiming to enhance registration accuracy in the case of partial visibility. We utilized four in vitro phantoms featuring realistic deformation and utilized a partial simulation pipeline to extract partial surfaces from five different regions encompassing different liver areas. The surface generation network can accurately generate the surface near the visible region, but the error is significant in areas far from the partially visible region. Furthermore, integrating the generated surface into registration revealed that the error affects registration outcomes not only in the invisible region, but also in the visible region in the camera's field of view.

Future research relying on a completion algorithm may utilize partial surfaces from multiple viewpoints. These partial observations from multiple views can be fused to create a more spatially complete point cloud before passing them into the surface completion network.

## Author Contributions

**Nakul Poudel**: conceptualization, methodology, experiment design and setup, investigation, formal analysis, validation, visualization, writing – original draft, writing – review and editing. **Zixin Yang**: conceptualization, methodology, experiment design and setup, data curation, writing – review and editing. **Richard Simon**: conceptualization, methodology, experiment design and setup, data curation, writing – review and editing. **Cristian A. Linte**: conceptualization, resources, funding acquisition, writing – review and editing, and supervision.

## Funding

The authors would like to acknowledge financial support from the National Institutes of Health (Award No. R35GM128877) and the National Science Foundation (Award No. CBET‐2245152). We also acknowledge access to computing support and resources provided by RIT Research Computing.

## Conflicts of Interest

The authors declare no conflicts of interest.

## Data Availability

The data that support the findings of this study are openly available in a GitHub repository at https://github.com/zixinyang9109/BCF‐FEM?tab=readme‐ov‐file.
